# Geospatialization of tuberculosis and income transfer programs among Indigenous peoples in an endemic territory

**DOI:** 10.1590/0034-7167-2022-0216

**Published:** 2022-11-28

**Authors:** Ingrid Bentes Lima, Laura Maria Vidal Nogueira, Lidiane de Nazaré Mota Trindade, Ivaneide Leal Ataide Rodrigues, Suzana Rosa André, Ana Inês Sousa

**Affiliations:** I Universidade do Estado do Pará. Belém, Pará, Brazil; II Universidade Federal do Rio de Janeiro. Rio de Janeiro, Rio de Janeiro, Brazil

**Keywords:** Tuberculosis, Government Programs, Health of Indigenous Peoples, Spatial Analysis, Geographic Information Systems., Tuberculosis, Programas de Gobierno, Salud de Poblaciones Indígenas, Análisis Espacial, Sistemas de Información Geográfica., Tuberculose, Programas Governamentais, Saúde de Populações Indígenas, Análise Espacial, Sistemas de Informação Geográfica.

## Abstract

**Objective:**

To analyze the spatial pattern of tuberculosis in Indigenous peoples from the State of Pará and its correlation with income transfer.

**Methods:**

Ecological study, with 340 cases reported in Indigenous peoples in the State of Pará, Brazil, in the period 2016-2020. The study performed a descriptive analysis and calculation of incidence rates with smoothing by the local empirical Bayesian method. The Global Moran index assessed the autocorrelation of the rates with income transfer data, *p*<0,05.

**Results:**

The Marajó and metropolitan mesoregions of Belém had the highest tuberculosis rates, and a reduced number of people benefited from income transfer (high-low correlation). The study identified high rates, and a significant number of people benefited from financial aid (high correlation high), I=0.399, p=0.027 in the Southwest.

**Conclusions:**

The spatial autocorrelation between tuberculosis and access to income transfer programs constitutes a relevant subsidy for the formulation of social protection policies and may impact the disease control actions in Indigenous territories, valuing the epidemiological heterogeneity identified in the mesoregions.

## RESUMO

**Objetivo:**

Analisar o padrão espacial de tuberculose em indígenas do Pará e sua correlação com transferência de renda.

**Métodos:**

Estudo ecológico, com 340 casos notificados em indígenas no Pará/Brasil, no período 2016-2020. Realizou-se análise descritiva e cálculo das taxas de incidência com suavização pelo método bayesiano empírico local. Fez-se autocorrelação das taxas com dados de transferência de renda pelo Moran Global, *p*<0,05.

**Resultados:**

As mesorregiões Marajó e Metropolitana de Belém apresentaram as taxas de tuberculose mais elevadas e reduzido número de pessoas beneficiadas com transferência de renda (correlação alto-baixo). No Sudoeste, identificaram se taxas elevadas e número significativo de pessoas beneficiadas com os auxílios financeiros (correlação alto alto), I=0,399, p=0,027.

**Conclusões:**

A autocorrelação espacial entre tuberculose e acesso a programas de transferência de renda constitui importante subsídio para formulação de políticas de proteção social, podendo impactar as ações de controle da doença nos territórios indígenas, valorizando a heterogeneidade epidemiológica identificada nas mesorregiões.

## RESUMEN

**Objetivo:**

Analizar patrón espacial de tuberculosis en indígenas de Pará y su correlación con transferencia de renta.

**Métodos:**

Estudio ecológico, con 340 casos notificados en indígenas en Pará/Brasil, entre 2016-2020. Realizado análisis descriptivo y cálculo de tasas de incidencia con moderación por el método bayesiano empírico local. Hecho autocorrelación de tasas con datos de transferencia de renta por Moran Global, *p*<0,05.

**Resultados:**

Las mesorregiones Marajó y Metropolitana de Belém presentaron las tasas de tuberculosis mayores y reducido número de personas beneficiadas con transferencia de renta (correlación alto-bajo). En el Sudoeste, identificaron tasas elevadas y número significativo de personas beneficiadas con auxilios financieros (correlación alto-alto), I=0,399, p=0,027.

**Conclusiones:**

La autocorrelación espacial entre tuberculosis y acceso a programas de transferencia de renta constituye importante subsidio para formulación de políticas de protección social, pudiendo impactar las acciones de control de la enfermedad en territorios indígenas, valorizando la heterogeneidad epidemiológica identificada en las mesorregiones.

## INTRODUCTION

Tuberculosis (TB) is considered multicausal and, among the individual and collective factors related to its occurrence, poor socioeconomic conditions can be favorable to the reproduction of the bacillus^(1-2)^. In addition, studies identified environmental conditions as influencing the worsening of epidemiological indicators, given the relationship with the social and economic pattern of the population, evidenced by the high rates of illness and mortality in developing countries. This scenario reflects social inequality and highlights the vulnerability of groups to the disease^(3)^.

From the perspective of Epidemiology, TB is influenced by population groups that have a higher risk of evolving from infection to active TB, called “special populations,” namely: immigrants; people living with the human immunodeficiency virus (HIV); people deprived of liberty; homeless population; Indigenous peoples; health professionals; users of alcohol and other drugs; smokers, and people with diabetes *mellitus* ^(3)^. This characterization was due to the unfavorable evidence in health and socioeconomic indicators in these population segments^(4)^.

At the global level, World Health Organization (WHO), concerned about reducing morbidity and mortality, has launched the United Nations Sustainable Development Goals (SDGs). Among the SDG targets for TB is to reduce mortality by 95% by 2035^(5)^. The General Coordination of the National Tuberculosis Control Program (CGPNCT), in line with the strategy *End* TB, Launched the National Plan to End Tuberculosis (PNCT), proposing for 2021-2025 more specific and operational actions aimed at accelerating progress to achieving the goals by 2035^(6)^. In addition, to strengthen the strategy, it also recommended expanding access to health services for the Indigenous population and investing in improving adherence to treatment^(6)^.

Indigenous health care is configured in a specific model established in the National Policy for Health Care of Indigenous Population (PNASPI), with primary care offered in the context of villages by Multidisciplinary Teams of Indigenous Health (EMSI), aiming to meet the cultural specificities of these peoples^(7)^. Therefore, it is in this context that these teams must carry out actions to identify, diagnose and treat cases.

According to the WHO, an estimated 5.8 million people fell ill, and 1.5 million died from TB in 2020 worldwide^(5)^. In Brazil, that same year, 68.7 thousand new cases were reported, which is equivalent to the incidence coefficient of 32.4 cases/100 thousand inhabitants. Concerning mortality, around 4.5 thousand deaths were registered in 2019^(6)^, with a coefficient of 2.2 deaths per 100,000 inhabitants. Given this, Brazil is in the group of 30 countries with a high TB rate, responsible for 87% of the total cases that occur in the world^(5)^.

In 2020, the State of Pará (PA) registered 3,735 new cases, which correspond to the incidence coefficient of 43 cases/100 thousand inhabitants; and 255 deaths, whose mortality rate was 3 cases/100 thousand inhabitants^(8)^. Contributing to these indices are populations more vulnerable to illness, such as Indigenous peoples, who in 2020 had an incidence coefficient of 150.9 cases/100 thousand inhabitants^(8-9)^, strengthening the thesis of the relationship between TB and social vulnerability^(3-4,10)^.

Despite government plans and actions aimed at greater TB control among Indigenous population, challenges remain due to the way of life, especially in villages, and social and health inequalities^(7)^. Therefore, it is necessary to implement policies to combat poverty with the effective participation of the community to promote access to services, respecting their social, economic, and cultural condition^(11)^.

The state, through social security, must implement policies and programs linked to social assistance^(12)^. In Brazil, social inequality and economic/financial difference declined when conditional cash transfer programs, mainly the Bolsa Família (a welfare stipend) program (PBF) and the Continuous Benefit program (BPC), were implemented and made available to the population^(12)^.

The study is relevant since there are gaps in scientific evidence related to the impact of government programs on tuberculosis indicators, especially in Indigenous populations^(11-13)^. National and international studies point to the scarcity of research addressing the problem, essentially focusing on the real needs of patients and their relationship with geospatial^(11,13)^.

Research related to social incentives to improve morbidity and mortality indicators is still incipient, especially in the Indigenous population. Nevertheless, scientific evidence reveals the importance of income transfer policies in combating poverty and reducing social inequality because of the link between the educational sector and health, representing a differential for the most vulnerable, such as Indigenous peoples^(14)^. To that end, the development of research that evaluates the impact of assistance programs, such as income transfer, may contribute to better TB control, aiming at achieving the goals of the strategy *End* TB^(15)^.

Even though the disease has been decreasing in the country, knowledge about the evolution of the epidemic and the reduction of morbimortality is necessary for the Indigenous population^(4)^. Therefore, the incorporation of spatial analysis techniques stands out as a tool to measure the spatial dependence of TB cases, using the Geographic Information System (GIS) and remote sensing^(16)^.

## OBJECTIVE

To analyze the spatial pattern of new cases of tuberculosis in Indigenous populations of the state of Pará and its correlation with income transfer.

## METHODS

### Ethical aspects

This study was developed with publicly available data on two government websites: the Department of Informatics of the Unified Health System (DATASUS), which also houses information on notification and closure of TB cases, and the Brazilian Institute of Geography and Statistics (IBGE). Thus, the ethical assessment was waived according to Resolution No. 510/16 of the National Health Council in its sole paragraph of Article 1.

### Study, design, and place of study

It is an ecological study, which adopted STROBE (EQUATOR) as a reference. It was conducted in the state of Pará from 2016 to 2020. Pará is in the Northern Region of Brazil, consisting of 144 municipalities distributed in 6 mesoregions: Marajó, Northeast Pará, Southwest Pará, Southeast Pará, Lower Amazonas, and the Metropolitan region of Belém^(17)^. It had an estimated population of 8,777,124 inhabitants in 2020, of which 111,142 are Indigenous people^(18)^. Mesoregion is the classification adopted by the IBGE that refers to the regional division that brings together municipalities with similar geographical and socioeconomic characteristics^(17)^.

### Population and selection criteria

The study considered new TB cases in Indigenous villagers as the population, reported in the studied period - between January 2016 and December 2020.

### Study protocol

The study obtained data on new TB cases from the Information System on Notifiable Diseases (SINAN) of the Brazilian Ministry of Health, made available by DATASUS. The selected variables were gender, age group, education level, year of notification, and the municipality of occurrence (later grouped into mesoregions). The search obtained the epidemiological data in May 2021 and organized it in spreadsheets in Microsoft Office Excel^®^ 2010 format. The data regarding the transfer of income to the Indigenous villagers were extracted from the IBGE 2010 Demographic Census; and the vector databases in the format *shapefile* with limits of the mesoregions of the State, which constitute the unit of analysis of the study, were also obtained from the IBGE website.

### Results analysis and statistics

The research grouped data according to the mesoregions of the state and applied descriptive statistics to set forth the social profile of new cases of Indigenous TB in each mesoregion. The study used the local empirical Bayesian method to minimize the instability of crude rates and to correct causal random fluctuations that occur primarily in municipalities with small numbers of inhabitants.

The calculations of the crude and smoothed rates were performed using the free statistical environment R version 4.0.3 and spatialized by TerraView version 4.2.2. The Qgis software version 3.20 produced the distribution maps of the local empirical crude and Bayesian incidence rates.

Finally, the Moran Global Index (I) performed bivariate analysis to obtain a spatial autocorrelation between TB incidence rates and the Indigenous population as beneficiaries of income transfer programs in each mesoregion of the state. For this, the hypothesis of “inverse” (I < 0), “randomness” (I = 0) and “direct” (I > 0) spatial autocorrelation was admitted, with significance of *p < 0,05*. It was considered strong spatial autocorrelation if the “I” was close to one of the limits [-1,., 1] considered a variation. The GeoDa software, version 1.14, spatialized the information.

## RESULTS

The study identified 340 new cases of TB and the corresponding incidence rate was 305.91/100,000 inhabitants. Concerning the Indigenous peoples registered in the income transfer assistance programs, there were 18,945 beneficiaries. Of the reported cases of TB, the majority were registered in 2019 (93/27.35%). There was a predominance in the age group of 20 to 39 years (117/34,41%) followed by 1 to 14 years (94; 27.65%), males (172; 50.59%), ignored schooling (90; 26.47), incomplete Elementary Education (83; 24.41%) and people without schooling (71; 20.88%) (Table 1).

**Table 1:** The social profile of tuberculosis cases in Indigenous peoples reported in the state of Pará, from 2016 to 2020, Belém, Pará, Brazil, 2022, N = 340

	n	%
Gender		
Male	172	50.59
Female	168	49.41
Age		
1 to 14	94	27.65
15 to 19	39	11.47
20 to 39	117	34.41
40 to 59	46	13.53
>= 60	44	12.94
Schooling		
No schooling	71	20.88
Incomplete Elementary School	83	24.41
Complete Elementary School	22	6.47
Incomplete High School	10	2.94
Complete High School	11	3.24
Incomplete Higher Education	4	1.18
Complete Higher Education	2	0.59
Ignored	90	26.47
Not applicable	47	13.82

*Source: DATASUS/TABNET (2020).*

**Figure 1:**
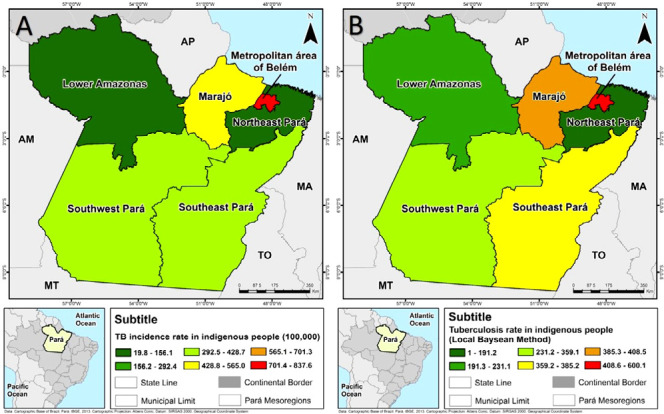
Spatial distribution of the crude Tuberculosis incidence rate (A) and rate smoothed by the local empirical Bayesian method (B) in the period from 2016 to 2020, Belém, Pará, Brazil, 2022

*Source: DATASUS / TABNET (2020) and IBGE (2010)*

The research identified higher TB coefficients in the Metropolitan mesoregion of Belém, whose gross incidence rate reached 837.61 cases/100 thousand inhabitants, and in the Marajó mesoregion, with 454.55 cases/100 thousand inhabitants. The Lower Amazon and the northeast of Pará presented the lowest figures, in the range of 19.8/100 thousand inhabitants and 156.1/100 thousand inhabitants (Figure 1.A).

Even after smoothing the rates, the spatial pattern of the disease remained, with the highest rates in the Metropolitan mesoregion of Belém, oscillating between 408.6 and 600.1/100 thousand inhabitants, and in Marajó, with coefficients between 385.5 and 408.5/100 thousand inhabitants, demonstrating an irregular pattern and random distribution of cases (Figure 1B).

**Figure 2:**
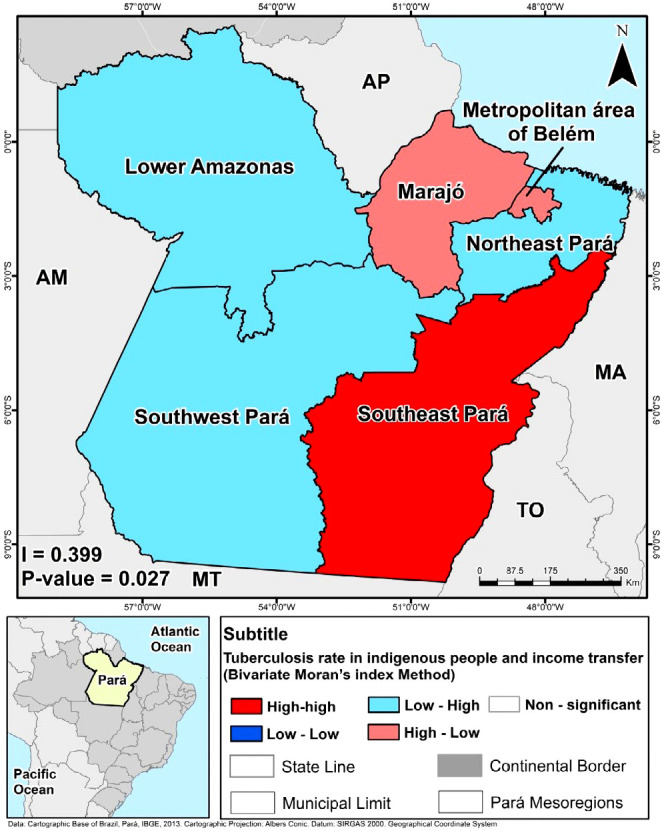
Bivariate Moran’s index of crude tuberculosis incidence rate and income transfer, Belém, Pará, Brazil, 2022.

*Source: DATASUS/TABNET (2020) and IBGE (2010)*

According to Figure 2, the Southeast mesoregion had a high proportion of beneficiaries of income transfer programs and high-high correlation with TB rates. Nevertheless, the mesoregions Northeast Pará, Lower Amazonas, and Southwest Pará had a low proportion of TB detection and a low proportion of Indigenous beneficiaries of income transfer programs.

The Marajó and Metropolitan of Belém mesoregions, which presented higher incidence rates (Figure 1B) and high-low correlation, are geographically close to mesoregions with a low proportion of cases. In addition, the research identified direct spatial autocorrelation (I = 0.399) and significant correlation between the variables studied (p = 0.027).

## DISCUSSION

The spatial dependence between TB and access to social benefits among Indigenous peoples points to the possibility of interethnic contact with sources of *Mycobacterium tuberculosis* infection because of the displacement of the villages to the urban centers, where the bank branches are located, to receive financial aid.

Indigenous populations, considered to be in a vulnerable situation, demand equitable health care and social protection. The latter aims at reducing economic and social inequalities and respecting ethnic and cultural profiles. Income transfer programs are strategies to seek social balance to ensure a decent standard of living. In Brazil, social benefits with the granting of cash to the most economically exposed people became a constitutional right in 1988, with the aim of promoting access to essential rights, goods, and services^(11 13)^.

Over the years, studies have observed that there was a variation in the number of cases, with a higher incidence in 2019. Although the WHO report and the Ministry of Health indicate a reduction in the incidence of TB in the general population since 2000, this rate among Indigenous people has remained stable in most Brazilian regions. Study^(4)^ conducted with Indigenous populations of Pará from 2005 to 2013 identified an incidence coefficient of 115.16/100 thousand inhabitants, a rate higher than the national average and the average of the State of Pará^(6,8)^.

It is admitted that the reduction in cases in 2020 may be due to the COVID-19 pandemic since there was a change in the routine of health services, with prioritization of pandemic control actions, thus compromising the diagnosis and treatment of TB. Therefore, possible underreporting of cases is not ruled out, and this is a concern, given that Indigenous peoples are a group at increased risk for TB disease, three times more than the general population^(3,10,19)^.

According to the guidelines for the prevention and control of TB in Indigenous populations of the region of the Americas, published by the Pan American Health Organization (PAHO), TB has been shown to be an aggravating factor in this population, with incidence estimates ranging from two to ten times higher in relation to non-Indigenous peoples^(20-21)^.

The behavior of the disease among Indigenous populations ratifies a higher proportion of cases in males, although with a slight difference in relation to females. Other studies evidenced such findings with attribution to possible difficulties of men attending health services for reasons of economic instability, precarious working conditions without the right to paid sick leave, prioritization of work at the expense of health, and the erroneous idea that seeking help is a sign of fragility^(19,22-23)^.

Additionally, there are cultural factors related to the difficulties of Indigenous men seeking health services, such as social gender norms, the perception that men need to show themselves strong to the family and community, demonstrating the ability to continue work and only seek assistance in advanced stages of illness^(23)^.

Still, on the social profile, the predominance of cases among Indigenous peoples with incomplete primary education followed by people who did not attend school ratifies the relationship of TB with low schooling. The study emphasizes that the level of education is decisive in understanding the mechanism of transmission of the disease and adherence to treatment, explaining the greater involvement of people with low school attendance^(22)^.

Concerning the higher proportion of cases among young adults, it may be due to the day-to-day permeated by intense mobility in the territory and contacting sources of infection. These are economically active people with work occupations and difficulties in missing work. In addition, they have precarious living conditions and the need to ensure family support, leading them to postpone the search for health services^(1)^.

The significant TB registration among people up to 14 years of age possibly refers to the demographic growth characteristic of the Brazilian Indigenous population, in which children and adolescents add significant quantitative, configuring a population pyramid with a broader base, different from the general population of Brazil^(18)^. Thus, the absolute number of cases may be directly related to the population size in the age group.

The spatial distribution of TB among Indigenous peoples in the State of Pará presented heterogeneity with a concentration of cases in the Marajó and Metropolitan mesoregions of Belém. Regarding the Metropolitan Area of Belém, it has an Indigenous Health Support House (CASAI) in the capital of Pará, which receives Indigenous peoples from various villages, especially from the Guamá-Tocantins Special Indigenous Health District based in Belém, to meet health needs, which may be having an impact on the high rate of cases reported in the mesoregion.

In Marajó, the high incidence rates may be influenced by the small local Indigenous population. A study conducted in this mesoregion, from 2013 to 2018, considering the general population, identified TB incidence ranging from 55.07/100 thousand inhabitants and 32/100 thousand inhabitants, thus, below the rate in Indigenous^(24)^. This data confirms the difference in the behavior of the disease between Indigenous and non-Indigenous peoples and strengthens the need to conduct studies with this group^(4)^.

The Marajó mesoregion, also known as the Marajó archipelago, comprises 16 municipalities and hosts the worst Municipal Human Development Indices (MHDI) in Brazil, ranging from 0.418 to 0.543, which reflects the situation of vulnerability and social exclusion of the population in general, both Indigenous and non-Indigenous. Studies point to health as a worrying factor in this mesoregion, as there is an insufficient number of beds, medical care, and health care, aggravated by the difficulty of access throughout the archipelago^(7,24)^. The behavior of TB in the mesoregion is still little addressed, being a territory neglected by the State^(24)^.

This study revealed a direct association between the occurrence of TB in Indigenous peoples and the receipt of social benefits, indicating that the higher the proportion of individuals benefiting from social programs, the higher the incidence rate of TB. It is a finding that may indicate a possible consequence of the displacement of Indigenous peoples from villages to city centers to receive cash from social benefits. In urban perimeters, contact with the general population intensifies with the possibility of infection by the *Mycobacterium tuberculosis* virus and subsequent TB illness^(14)^.

Nevertheless, a study that evaluated the incidence of TB and BFP coverage in five thousand Brazilian municipalities from 2004 to 2012 in the general population concluded that there was a reduction in the incidence rate of the disease from 46.1 to 38.8/100 thousand inhabitants, proportional to the increase in the coverage of the benefit^(14)^. Thus, it is necessary to evaluate the factors intrinsic to the management of the subsystem of Indigenous health care and the policies of management and transfer of social benefits to Indigenous peoples.

It is challenging to establish or even adapt health care and social protection strategies for Indigenous populations, given the peculiarities that involve the way of life, the culture, the histories of illness, the dynamics of the territory, and the difficulties of displacement. In addition to the challenges presented, initiatives external to Amerindian interests, such as the implementation of large power generation projects, agricultural expansion, and logging enterprises, have produced intense social, economic, and environmental transformations since the 1970s. Such initiatives have mainly reached the southeast and Southwest mesoregions municipalities, which have the largest Indigenous population contingent in the State of Pará^(24)^. Even in the face of economic investments, much progress must be made to achieve improvement in the quality of life of Indigenous peoples. The limitation of access to health services and social exclusion are aspects that deserve attention and must be prioritized in the agenda of Indigenous interests^(4,11,22)^, as they directly impact the standard of living. The access to essential services by Indigenous peoples depends on technical-operational, geographical, financial, and linguistic factors^(7)^.

Public policies are a form of intervention in response to a situation considered problematic in society and should generate impacts on the target audience^(25)^. In the context of severe inequality, social programs and projects minimize inequalities and reduce poverty, even if they fall short in generating opportunities in the labor market^(11,26)^. Thus, the topic deserves further investigation.

The study considers that TB in Indigenous peoples is not related only to the association between illness and the receipt of social benefits. It is necessary to consider other aspects that generate health inequities, such as difficulty in accessing health services, difficult geographical location, low education, land expropriation, and loss of historical and cultural identity^(7)^.

Tackling TB in Indigenous populations requires a differentiated approach, considering the social context of precarious living conditions, interculturality, and beliefs of these populations^(20)^. In this context, nursing plays a relevant role in prevention, control, diagnosis, and treatment, through the adoption of strategies aimed at respecting local culture, breaking stigmas, differentiated health education, and adequate management of health services.

### Study limitations

As limitations of the study, the study considered a possible underreporting of cases, incompleteness of TB data in Indigenous peoples, and lack of information on income transfer in certain municipalities. In this respects, researchers have endeavored to obtain all available data, making successive consultations in the storage sources, especially data related to income transfer.

### Contributions to the field of Nursing

The findings may contribute to the recognition of the areas with the highest occurrence of TB among Indigenous peoples in the state of Pará and support the development of action plans aimed at the locoregional epidemiological reality. They can also guide intersectoral policies to adopt more equitable strategies in the granting of social benefits of income transfer and, thus, contribute to improving people’s quality of life.

## CONCLUSION

The fight against TB among Indigenous peoples is challenging and must be added to social protection policies because it is a disease that affects natives more significantly than the general population. The relationship between TB and poverty further exacerbates the spread among Indigenous populations, whose social and economic profile is suffering. Thus, it is necessary to establish strategies for differentiated financial assistance to assist families with people undergoing treatment for TB and thus combat the disease, especially in the context of villages.

Access to BFP harmed TB incidence rates, which points to the need to review the format of income transfer programs for Indigenous populations. It is necessary to observe the local culture, given the strong spirit of collective sharing that permeates daily life in the villages, causing the money destined for a particular person or age group to be reverted to the whole family, compromising its end.

The financial vulnerability of Indigenous populations requires careful analysis of the conditions of income transfer to reduce not only hunger, poverty, and malnutrition, but also to strengthen social protection policies, aiming to achieve quality education, better food standards, greater access to health services, and other income-generating opportunities. In this context, an intersectoral approach in the management of health services to Indigenous peoples should be promoted since social protection strategies can contribute to the achievement of methods for better TB control.
